# Fresh 3D Printing of Spanlastics Hydrogel for Drug Delivery Applications *In Vitro*

**DOI:** 10.1007/s11095-026-04068-6

**Published:** 2026-03-06

**Authors:** Elom Doe, Abigail Alabi, Leela Raghava Jaidev Chakka, Mohammed Maniruzzaman

**Affiliations:** 1 Pharmaceutical Engineering and 3D Printing Lab (PharmE3D), Department of Pharmaceutics and Drug Delivery, School of Pharmacy, University of Mississippi, 1 University Avenue, University, MS 38677 USA; 2 Department of Chemical and Biomolecular Engineering, University of Maryland, College Park, MD 20742 USA

**Keywords:** 3D printing, anti-cancer, FRESH, hydrogels, *in vitro* therapeutics, localized drug delivery, MCF7, spanlastics

## Abstract

**Purpose:**

To develop a localized delivery implant by integrating doxorubicin loaded spanlastic vesicles within Freeform Reversible Embedding of Suspended Hydrogels (FRESH) printed alginate constructs.

**Methods:**

Spanlastics composed of Sorbitan Monostearate (Span60) and an edge activator, Polyethylene sorbitol ester (Tween 80), were prepared by ethanolic injection. Plain and drug-loaded spanlastics were characterized for their physicochemical properties. Vesicles were incorporated into 3D printed sodium alginate hydrogels in ‘FRESH’ bioprinting process to promote the sustained drug release of doxorubicin which was assessed using dialysis membrane for drug release. *In vitro* uptake and cytotoxicity were evaluated in MCF7 breast cancer cells.

**Results:**

Optimized formulations produced vesicles of approximately 200 to 300 nm with moderate encapsulation efficiency (33 to 44%) and stability during hydrogel incorporation and printing. Printed depots provided sustained doxorubicin release relative to suspension and reduced MCF7 viability, with preferential intracellular and nuclear localization consistent with doxorubicin activity.

**Conclusion:**

Spanlastic-loaded FRESH printed alginate implants combine vesicle-mediated cellular delivery with matrix-governed sustained release, supporting their potential as a localized chemotherapy depot for further *in vivo* validation.

## Introduction

Systemic chemotherapy is constrained by narrow therapeutic windows, dose-limiting toxicities, and heterogeneous drug exposure across tumor microenvironments [1, 2]. Optimum therapeutic activity is not achieved due to non-specific distribution, efflux-mediated resistance, among others [3–6]. There is a pressing need for localized, engineered drug depots that can concentrate payloads at disease sites, modulate release profiles, and navigate complex biological barriers without compounding systemic burden [7–9].

Spanlastics are ultra-deformable vesicles composed of a nonionic surfactant, typically a Span, together with an edge activator [10]. The edge activator lowers interfacial tension and increases bilayer elasticity, which confers high deformability, improves membrane permeation, and may lessen the impact of efflux mechanisms [11, 12]. These vesicles can encapsulate both hydrophilic and lipophilic drugs. In ocular, dermal, and mucosal models, they have shown greater transport and bioavailability than conventional niosomes and related carriers, which is especially useful for poorly soluble phytochemicals and labile small molecules [13, 14]. Preparation is well established through thin-film hydration, ethanol injection, microfluidization, or sonication, and each method can be tuned to achieve the desired particle size, polydispersity, and encapsulation efficiency [12, 14–16].

Beyond their definition, spanlastics are distinguished by the functional role of the edge activator in modulating membrane elasticity and interfacial behavior. Compared with more rigid nonionic vesicles and conventional bilayers, this elasticity is frequently linked to improved interaction with biological interfaces and enhanced transport across barrier like tissues in topical and ocular settings, where spanlastic formulations have repeatedly been used to increase local delivery and apparent bioavailability relative to comparator vesicular systems [10, 11, 17]. From a formulation standpoint, spanlastics also offer a practical design space because the Span to edge activator ratio provides a direct handle to tune drug retention, membrane permeability, and release behavior without introducing complex chemistries. This is particularly relevant for localized depots, where early burst and diffusion driven loss from hydrated matrices can limit performance for freely soluble drug. Embedding deformable vesicles within a hydrogel can introduce a second level of control by coupling carrier governed drug association with matrix governed transport, which is conceptually well aligned with a printed alginate implant produced by FRESH, where geometry further shapes diffusion path length and release kinetics [18, 19].

Translating the advantages of these vesicles into spatially precise and clinically relevant depots calls for manufacturing methods that protect vesicle integrity while yielding delicate, high-fidelity constructs [19]. Freeform Reversible Embedding of Suspended Hydrogels (FRESH) allows extrusion of soft hydrogel inks inside a granular gelatin support bath, producing complex three-dimensional geometries that are released by gentle thermal liquefaction of the support [18, 20–22]. This approach provides a low-temperature, shear-managed environment that is compatible with bioactive cargos and soft matrices, conditions that are likely to help maintain spanlastic size, polydispersity, and encapsulation during printing [12, 16, 23]. FRESH printing provides a practical route to convert nanovesicle science into precise, implantable therapy, enabling the printing of soft hydrogels within a reversible gelatin support and gentle recovery of complex geometries [18]. Depots constructed in this way exhibit defined filaments, controlled pores, and patient-tailored shapes that seat securely at the target site and establish predictable diffusion paths [8, 18]. Spanlastic vesicles are incorporated as part of the matrix within these architectures instead of free drug, drawing on their documented deformability and membrane-permeation advantages in ocular and mucosal models [10, 12]. Geometry then governs transport, as filament diameter, infill pattern, and path tortuosity set local concentration gradients and release rates.

In the present study we hypothesize that FRESH-printed alginate hydrogels embedding an optimized spanlastic formulation will preserve vesicle integrity and drug compatibility, maintain the shear-thinning and recovery behavior required for high-fidelity printing, and deliver geometry-dependent sustained release that outperforms both free drug and non-printed spanlastic suspension, with improved penetration and cytotoxic efficacy over conventional controls.

## Materials and Methods

Doxorubicin hydrochloride, Span 60, Tween 80, sodium alginate, ethanol, phosphate-buffered saline (PBS, pH 7.4), formaldehyde, TritonX-100, Alamar blue assay, Gelatin (Type A), CaCl_2_ solution, were purchased from Sigma Aldrich, St. Louis, Missouri, USA. The snakeskin dialysis tubing of molecular weight cut-off (MWCO) 10 kDa, Alexaflor-phalloidin 488 dye, DAPI, were purchased from Thermo Scientific, Waltham, Massachusetts, USA. The breast cancer (human origin, MCF7) cells were procured from ATCC, Manassas, Virginia, USA. Dulbecco’s modified Eagle medium (DMEM), Fetal Bovine Serum (FBS), Penicillin–streptomycin, Trypsin–EDTA, were purchased from Gibco, Grand Island, NewYork, USA. 24 and 96 well plates were bought from CellTreat, Ayer, Massachusetts, USA, while the 6-well plate was from Nunc transwell, Corning, Corning, New York, USA. All materials were of analytical grade, procured and used without any changes unless otherwise mentioned.

### Preparation of Spanlastics

The solvent injection method was employed to prepare Spanlastics particles using Span 60 and Tween 80. A specified amount of Span 60 was dissolved in ethanol at 60–70°C. The ethanolic mixture was added dropwise into the aqueous phase under continuous stirring at 900 rpm at 60–70°C. The stirring of the suspension was continued for 60 min. The formulation was allowed to cool on the benchtop to ambient temperature (20–25°C) before further processing. The particles were centrifuged at 10,000 rpm for 15 min. The supernatant was separated, and the pellet (spanlastics), redistributed into milliQ water. The blank nanoparticles were labelled as Span.

### Preparation of Drug-Loaded Spanlastics

The drug Doxorubicin of pharmaceutical grade was used for this study. 5 mg per 10 mL (0.5 wt% (w/v)) and 10 mg per 10 mL (1 wt% (s/v)) of Dox were added to the aqueous phase during the preparation of the spanlastics. The Dox-loaded spanlastics were labelled as Span-Dox-0.5 and Span-Dox-1.0, respectively.

### Encapsulation Efficiency/Drug Loading

The drug loading in the nanoparticles was evaluated indirectly by calculating the free/non-encapsulated drug in the supernatant after centrifugation. The free drug was quantified after dilution and extrapolation of absorbance to determine concentration on a standard calibration curve using a UV–visible spectrophotometer at 485 nm (BioTek, Winooski, Vermont, USA). The total percentage of drug encapsulation was calculated using the formula: [(Total Drug-Free Drug in mg)/Total Drug (mg)] *100.

### Characterization

#### Particle Size Analysis

The particle size, polydispersity index, and zeta potential of spanlastics and drug-loaded spanlastics were measured by dynamic light scattering (Zetasizer Nano ZS, Malvern Panalytical, Kassel, Germany). Prepared dispersions were diluted in filtered deionized water to an appropriate scattering intensity to minimize multiple scattering, gently inverted, and equilibrated at 25°C for 5 min before analysis. Particle size is reported as Z-average diameter, and the polydispersity index was recorded as a measure of size distribution. For size measurements, samples were loaded into disposable polystyrene cuvettes, while zeta potential measurements were performed using folded capillary cells. Measurements were conducted in triplicate.

#### Scanning Electron Microscopy (SEM)

The morphology of spanlastics was examined using scanning electron microscopy (JSM 5600, JEOL Ltd., Akishima, Tokyo, Japan). Samples were pre-fixed, dried, and mounted on 147 aluminum SEM stubs using double-sided adhesive conductive carbon tape, and sputter-coated 148 with 10 nm layer of gold–palladium (60:40) using a Desk V HP sputter coater (Denton Vacuum, 149 Moorestown, New Jersey, USA) under an argon atmosphere to render the surface conductive. 150 Imaging was performed at an accelerating voltage of 10 keV with a working distance of 6 mm. 151 The micrograph was acquired at 50,000 × magnification. Quantitative measurements were 152 conducted using the instrument’s integrated image analysis software.

#### Fourier Transform Infrared Spectroscopy (FTIR)

Fourier transform infrared spectroscopy was performed using an FTIR spectrometer (Equinox 55, Bruker Optics GmbH & Co. KG, Ettlingen, Germany) operated in attenuated total reflectance mode. Doxorubicin hydrochloride, individual excipients (Span 60, Tween 80, sodium alginate), and the final formulations were analyzed. For ATR measurements, samples were placed directly onto the ATR crystal and pressed to ensure uniform contact. Spectra were collected over 4000 to 400 cm⁻^1^ with a spectral resolution of 4 cm⁻^1^ and 32 scans per sample. Background spectra were acquired prior to each measurement and automatically subtracted.

#### Differential Scanning Calorimeter (DSC)

Thermal transitions of unloaded and doxorubicin-loaded spanlastics were evaluated using a differential scanning calorimeter (DSC Q20, TA Instruments, New Castle, DE, USA). Weighted samples (5–7 mg) of doxorubicin hydrochloride, sodium alginate, Span 60 and freeze-dried samples of unloaded and drug-loaded spanlastics were sealed in aluminum DSC pans, with an empty sealed pan used as the reference. Measurements were performed from 25 to 300°C at a heating rate of 10°C per min under a nitrogen purge of 50 mL per min. Thermograms were analyzed to identify endothermic and exothermic events and shifts in thermal transitions.

### Drug Release and Release Kinetics

The Span-Dox nanoparticles and Span-Dox Hydrogel each containing 5 mg of drug (*n* = 3 for each group), were loaded into a dialysis bag made from the snakeskin dialysis tubing, (MWCO 10 kDa) and immersed in PBS, pH 7.4, for 120 h. Aliquots of 1 mL were collected at various time points, and the drug content was measured using a UV–visible spectrophotometer at 485 nm (BioTek, Winooski, Vermont, USA). The cumulative drug release was calculated and recorded.

### Cell Culture

The breast cancer (human origin, MCF7) cells were cultured in Dulbecco’s modified Eagle medium (DMEM) supplemented with 10% foetal bovine serum (FBS) and 1% Penicillin–streptomycin. The cells were incubated in a CO_2_ incubator (Cellexpert, Eppendorf, Framingham, Massachusetts, USA) at 37°C with 5% CO_2_. The cells at 80% confluency were trypsinized using 0.25% Trypsin–EDTA for 3 min. The activity of trypsin was neutralized by adding media to the flask. The cells were centrifuged at x200g (Eppendorf, USA) for 3 min. The cell pellet was redispersed in media and counted using CountessII (Life Technologies, Waltham, Massachusetts, USA).

### Cell Uptake

The MCF7 cells were counted to achieve 5 × 10^4^ cells per well per 1 mL media. The cells were allowed to attach in a 24-well plate for 24 h. The Span-Dox1.0 nanoparticles of 0.25 mg/mL of 100 µL was added to 1 mL of media per each well for the cells for 4 h. At each time point of 30 min, 1, 2, and 4 h, the cells were fixed using 4% formaldehyde in PBS (pH 7.4) for 30 min. The cells were washed twice with PBS. The cells were permeabilized with 0.1% TritonX-100 for 10 min. The cells were washed twice with PBS. The Alexaflor-phalloidin488 dye (1 µg/mL) was added to the cells, incubated for 1 h at room temperature in the dark to stain the actin filaments of the cells. The nucleus was stained using DAPI (1 µg/mL) for 15 min. The cells were imaged using a fluorescence microscope (IX83, Olympus, Colorado Springs, Colorado, USA) using the filters green for actin staining, blue for the nucleus, and red for Dox. The images were taken at 20 × magnification. The final images exported as.jpg were processed to merge the channels using ImageJ (v1.54 g, ImageJ).

### Evaluation of Anti-Cancer Activity of Dox-loaded Nanoparticles

The MCF7 cells were counted to achieve 1 × 10^4^ cells per well per 100 µL media. The cells were allowed to attach to the 96-well plate for 24 h. The Span and Span-Dox (0.5 and 1.0) nanoparticles at different dilutions from the stock of 5 mg Dox equivalent nanoparticles were UV sterilized in the biosafety cabinet (BSL2, Airstream, ESCO Lifesciences, Horsham, Pennsylvania, USA). The nanoparticles were reconstituted to 1 mL (stock) and diluted to get the final amounts of 0.5, 0.25, 0.025, 0.0125, & 0.00125 mg/mL, using cell media. The spent media from the well plate was removed, and 100 µL of the Span and Span-Dox groups was added to each well. The plate was incubated for 24 h. The viability was evaluated using the Alamar blue assay. After incubation, the media was removed, and 10 µL of alamar blue with 90 µL of cell media was added to each well and incubated for 4 h. The color change in each well was read at 560/590 fluorescence in a well plate reader (BioTek, Winooski, Vermont, USA).

### FRESH 3D printing of Span and Span-Dox Hydrogels

#### Preparation of FRESH

The 5% (w/v) Gelatin (Type A) was prepared in milliQ water at 50°C until gelatin dissolved. The solution was transferred to a mason jar and cooled overnight at 4°C. The crosslinking solution, 100 mM (0.1 M) CaCl_2_, was prepared and cooled at 4°C overnight. The mason jar was filled with CaCl_2_ solution and blended for 30 s. The gelatin slurry was centrifuged at x2000g for 5 min. The pellet was washed twice with CaCl_2_ solution and stored at 4°C until further use.

#### Preparation of Alginate Hydrogel (2% and 5% w/v)

Sodium alginate was weighed and dissolved in 100 mL of sterile distilled water in a 250 mL beaker (2 g and 5 g, respectively). The solution was stirred on a magnetic stir plate for 1–2 h until fully dissolved (clear, viscous). The hydrogel was sterilized by filtering through a 0.22 µm syringe filter in a biosafety cabinet.

#### Preparation of Span and Span-Dox hydrogels

The 5 mg Dox equivalent Span and Span-Dox1.0 nanoparticles were mixed with 5 wt% alginate hydrogel, respectively, which was prepared in a biosafety cabinet.

#### FRESH 3D Printing

A 3D computer-aided model of a 10 × 2 mm (diameter x height) disc was prepared and exported as an.stl file using Freecad (v1, USA). The.stl file was exported into the Bioprint Pro software (Allevi, 3D systems, Rock Hill, South Carolina, USA) along with the following parameters- extrusion pressure (8 kPa), speed (12 mm/s), and layer height (0.2 mm). The CaCl_2_ gelatin slurry-filled 6-well plate was placed on the bioprinter bed. The Span and Span-Dox1.0 hydrogels were loaded into the 5 mL syringes of the printer-compatible syringes and nozzles (25G, Allevi, 3D systems, Rock Hill, South Carolina, USA) and printed using the above parameters at room temperature. The printing was carried out under sterile conditions in the biosafety cabinet. After printing, the plate was kept in a 37°C incubator for 10–15 min to melt the gelatin slurry. The 3D printed hydrogels were washed gently with sterile PBS (2–3 times) to remove residual gelatin.

#### Transwell-assisted Anticancer Activity

The MCF7 cells were counted to achieve 1.5 × 10^5^ cells per well per 2 mL media. The cells were allowed to attach in a 6-well plate for 24 h. The FRESH 3D printed hydrogels of Span and Span-Dox1.0 were transferred to transwells in a 6-well plate. The transwells were provided with 2 mL of media, and the wells with cells were filled with 3 mL of media. The plate was incubated for 24 h in a CO_2_ incubator (Cellexpert, Eppendorf, Framingham, Massachusetts, USA) at 37°C with 5% CO_2_. The viability was evaluated using the Alamar blue assay (Sigma Aldrich, St. Louis, Missouri, USA). After incubation, the media was removed, and 100 µL of alamar blue with 900 µL of cell media was added to each well and incubated for 4 h. The colour change in each well was read at 560/590 fluorescence in a well plate reader (BioTek, Winooski, Vermont, USA).

#### Statistics

All experiments were run with groups in triplicate, and the data was represented as mean ± standard deviation. The statistical significance of difference, *p < 0.05 was done using one-way or two-way ANOVA with post Tukey test.

## Results

### Preparation of Spanlastics and Dox-loaded Spanlastics

**Fig. 1:**
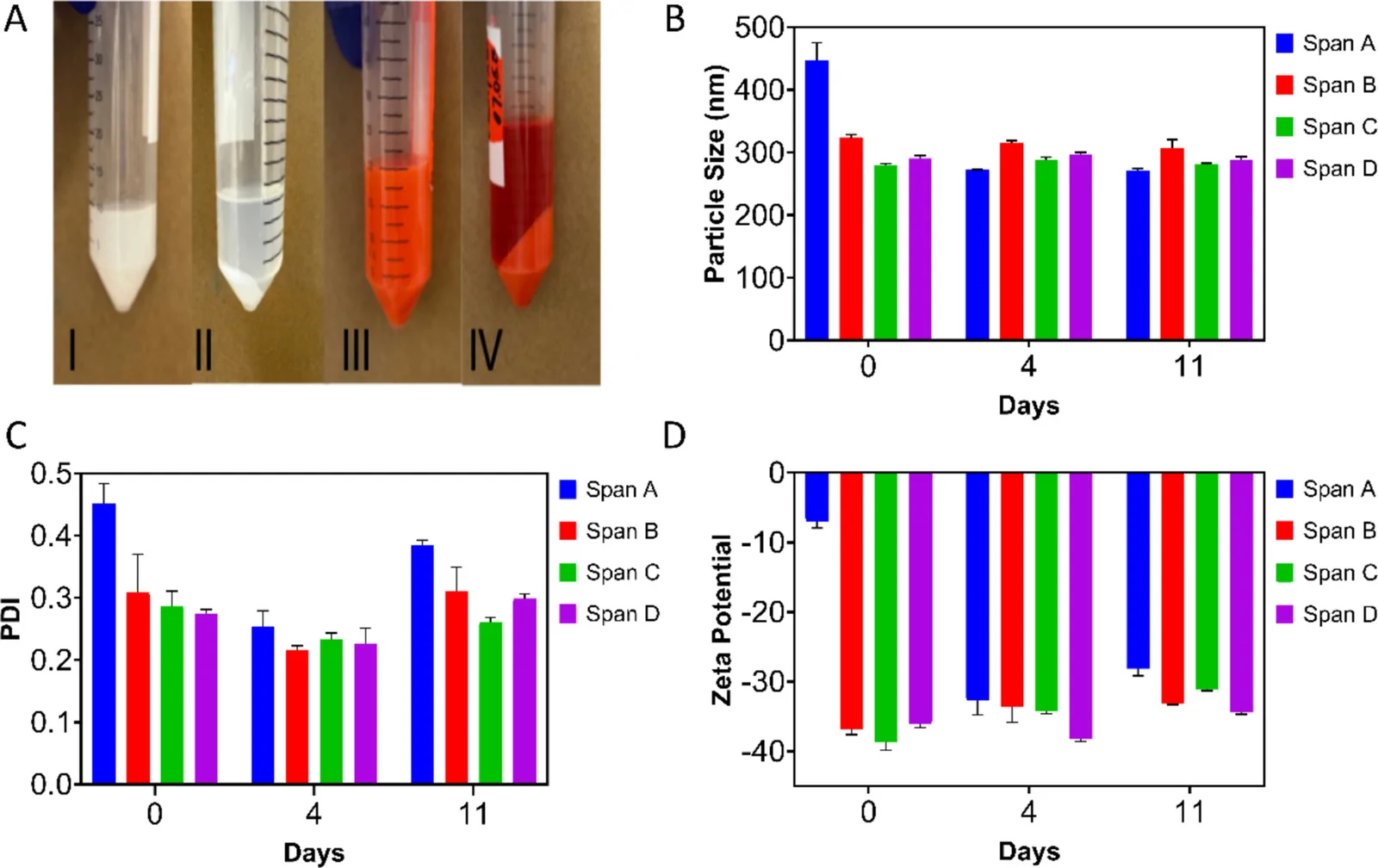
The synthesized nanoparticles and particle size analysis of spanlastics (Span) and doxorubicin-loaded spanlastics (Span-Dox). (**A**) optical images of the spanlastics before (I) and after centrifugation (II), showing the nanoparticle pellet, and the same for Dox-loaded spanlastics before (III) and after (IV) centrifugation. (**B**) Particle size analysis of the nanoparticles on different days (0, 4, and 11 days) after synthesis, showing the stability of the particles without any significant aggregation, with no significant increase in size. (**C**) Zeta potential of the span and Span-Dox nanoparticles (**D**) PDI of the Span and Span-Dox nanoparticles. All samples were run in triplicate, and the data were represented mean ± standard deviation. The statistical significance of the difference, *p < 0.05, was determined using one-way or two-way ANOVA with post Tukey test.

Varying ratios of Span 60 and Tween 80 were formulated by ethanolic injection of Span in ethanol into aqueous phase while altering the input of the Tween 80 in either ethanol or water, as seen in Table I. The formulation that was selected for drug loading was based on particle size characteristics, and the effect of Tween in ethanol or water. The synthesis of the Span and Span-Dox nanoparticles carried out using the solvent injection method yielded a particle size in the 260–430 nm range. The images in Fig. 1A show the synthesized and centrifuged Span and Span-Dox nanoparticles, yielding a pellet of nanoparticles. Figure 1B shows the particle size of the synthesized nanoparticles of around 300 nm, and the size remains constant for most formulations over 11 days, showing the stability of the particles and robustness of the synthesis procedure for spanlastics. Figure 1C and 1D show the polydispersity index (PDI) and zeta potential (ZP) of the Span and Span-Dox nanoparticles, showing a moderate distribution of the particles with the PDI values around 0.2–0.3, and ZP around −33 mV for most formulations.

**Table I:** Formulation Composition of Spanlastic Nanovesicles

Formulation	Span 60: Tween 80 (w/w)	Ethanol: Deionized Water (v/v)	Tween added to
Span A	70:30	1:3	Ethanol
Span B	70:30	1:3	Water
Span C	80:20	1:3	Ethanol
Span D	80:20	1:3	Water

Drug-loaded Spanlastics were formulated using drug concentrations of 0.5% w/v and 1.0% w/v for Span B and Span D, respectively. The process was optimized to achieve the desired particle size in the nanometer range. The formulation that was able to pelletize after centrifugation was selected for further analysis, as seen in Fig. 1a III and IV.

**Fig. 2:**
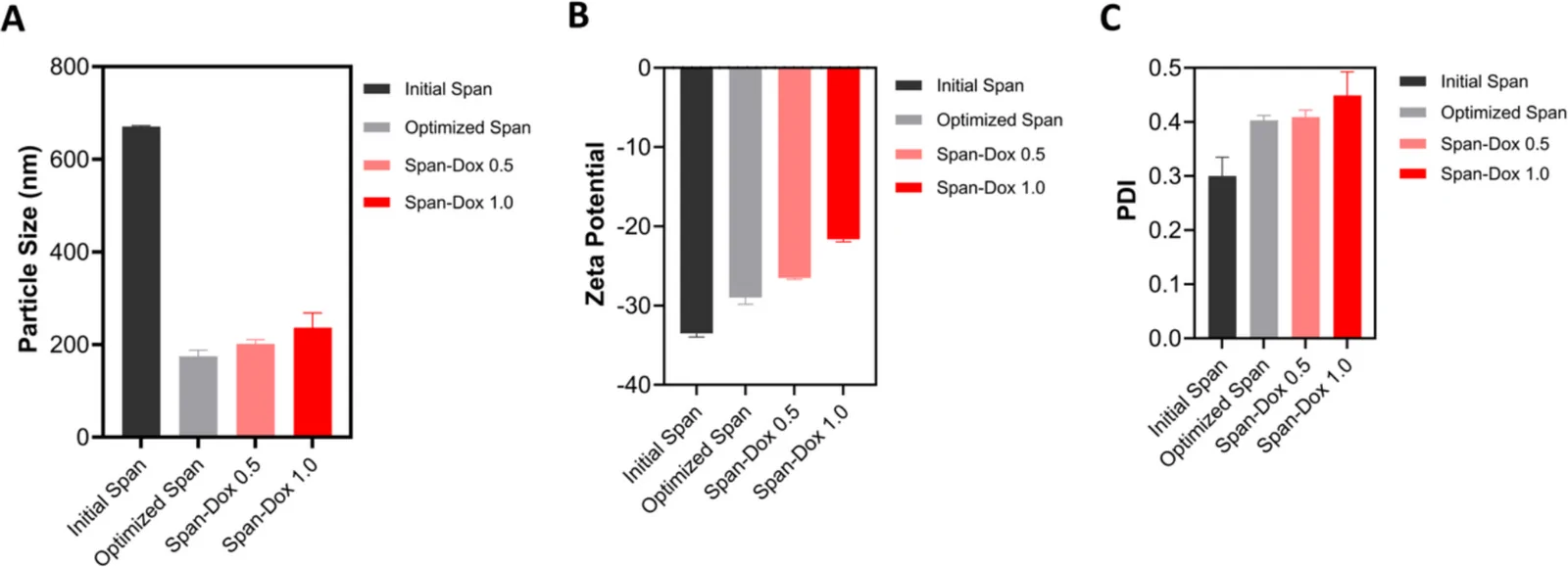
Plain spanlastics were formulated to optimize suitable nanoparticles, which were then drug-loaded with doxorubicin. An overview of the particle size (**A**), zeta potential (**B**), and PDI (**C)** of the initial formulation, optimized formulation, and drug-loaded Span-Dox. All samples were run in triplicates, and the data was represented mean ± standard deviation. The statistical significance of difference, *p < 0.05 was done using one-way or two-way ANOVA with post Tukey test.

Figure 2 shows the particle size, zeta potential, and PDI of span-Dox 0.5 and span-Dox 1.0, where the addition of Dox increased particle size while reducing the zeta potential, with PDI having no significant difference between span-Dox 0.5 and 1.0 groups.

### Encapsulation Efficiency

The encapsulation efficiency of Span Dox-0.5 and Span Dox-1.0 was quantified after separation of free drug from Spanlastics loaded with drug by ultracentrifugation. The calculation was based on the formula mentioned in Section " Encapsulation Efficiency/Drug Loading", with a mean recovery of the total drug of 40% percent. Across the Span 60 and Tween 80 ratios screened, EE% ranged from 33% ± 2 to 45 ± 1.5 with corresponding DL% from 0.17% ± 0.01 to 0.45% ± 0.02 (*n *= 3). The selected formulation (Dox1) achieved EE% of 45% ± 1.5 and DL% of 0.45% ± 0.02.

### Rheological Properties of Sodium Alginate Hydrogel

**Fig. 3:**
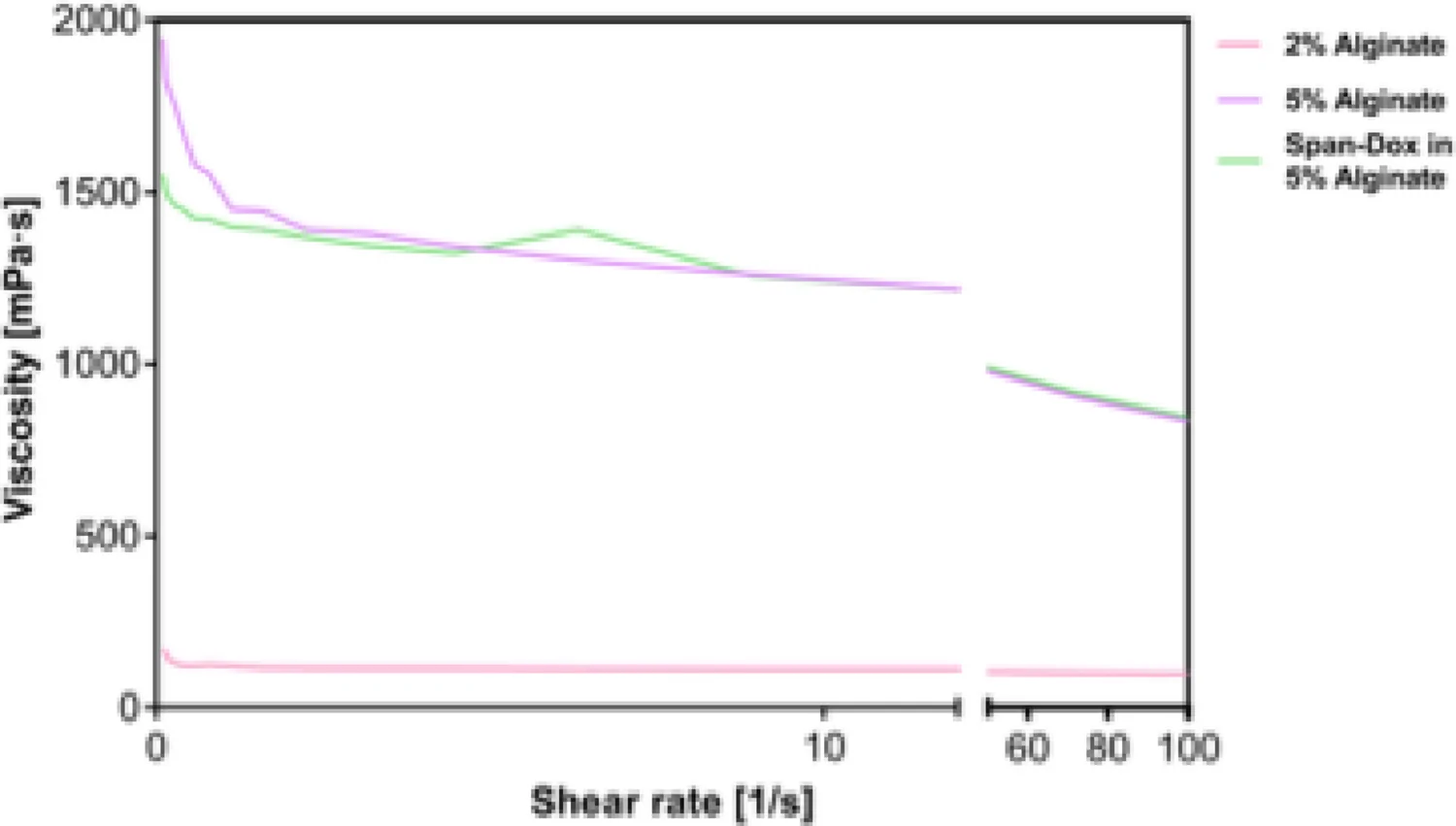
Viscosity flow curve of 2% and 5% (w/v) Alginate and Span-Dox loaded in 5% Alginate showing increased viscosity with increased concentration of Alginate and shear-thinning behaviour with increasing shear.

The viscosity of all formulations decreased with increasing shear rate from 0.1 to 100 s⁻^1^, confirming shear-thinning behavior suitable for semi-solid extrusion in 3D printing, as seen in Fig. 3. Across the full range, 5% sodium alginate showed the highest viscosity (about 2.0 × 10^3^ to 1.3 × 10^3^ mPa·s). Incorporating Span Dox vesicles into 5% alginate yielded intermediate values (about 1.5 × 10^3^ to 0.9 × 10^3^ mPa·s), lower than plain 5% alginate but higher than 2% alginate. The 2% alginate hydrogel exhibited the lowest viscosities throughout (about 1.7 × 10^2^ to 1.0 × 10^2^ mPa·s) with a comparatively flatter flow curve.

### Physicochemical Characterization of Spanlastics

#### FTIR

**Fig. 4:**
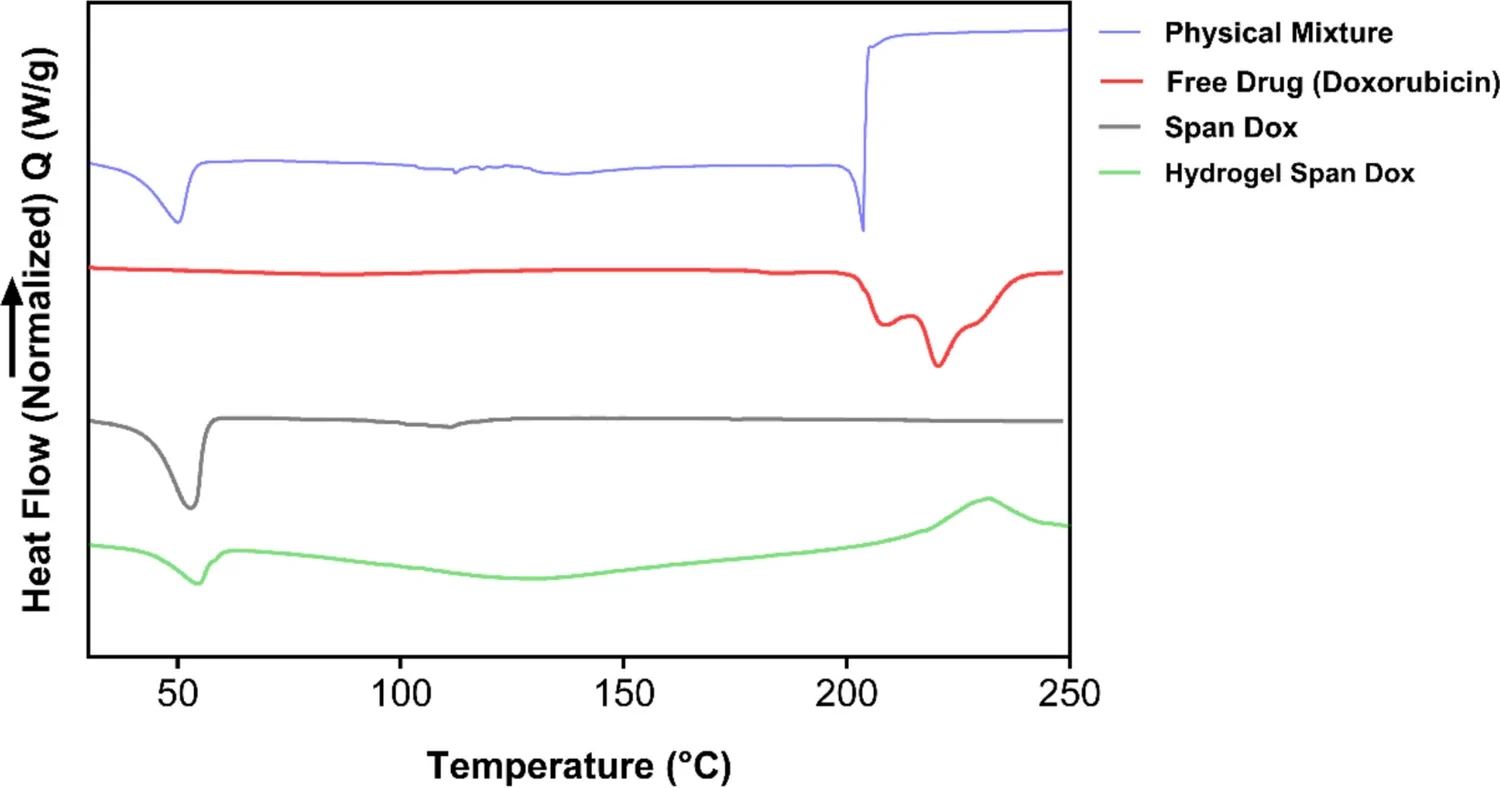
Depicts the FTIR spectra for free drug (Doxorubicin), Span 60, sodium alginate, and sodium alginate hydrogel loaded with Span-Dox.

The FTIR spectra, as seen in Fig. 4, showed the characteristic bands of free doxorubicin, Span 60, sodium alginate, and the hydrogel systems. Free doxorubicin displayed –OH/N–H stretching (~ 3400 cm⁻1), aromatic/aliphatic C–H stretching (~ 2920–2850 cm⁻1), quinone C = O stretching (~ 1720 cm⁻1), aromatic C = C vibrations (~ 1610–1580 cm⁻1), and C–O–C stretching (~ 1200–1000 cm⁻1) [24]. Sodium alginate exhibited broad –OH (~ 3400 cm⁻1), asymmetric COO⁻ (~ 1600 cm⁻1), symmetric COO⁻ (~ 1410 cm⁻1), and glycosidic C–O–C (~ 1020–1080 cm⁻1) [25, 26]. The hydrogel-loaded Span-Dox retained the alginate backbone bands, whereas drug-related carbonyl and aromatic signals were markedly reduced or overlapped with alginate features [27, 28].

#### DSC

**Fig. 5:**
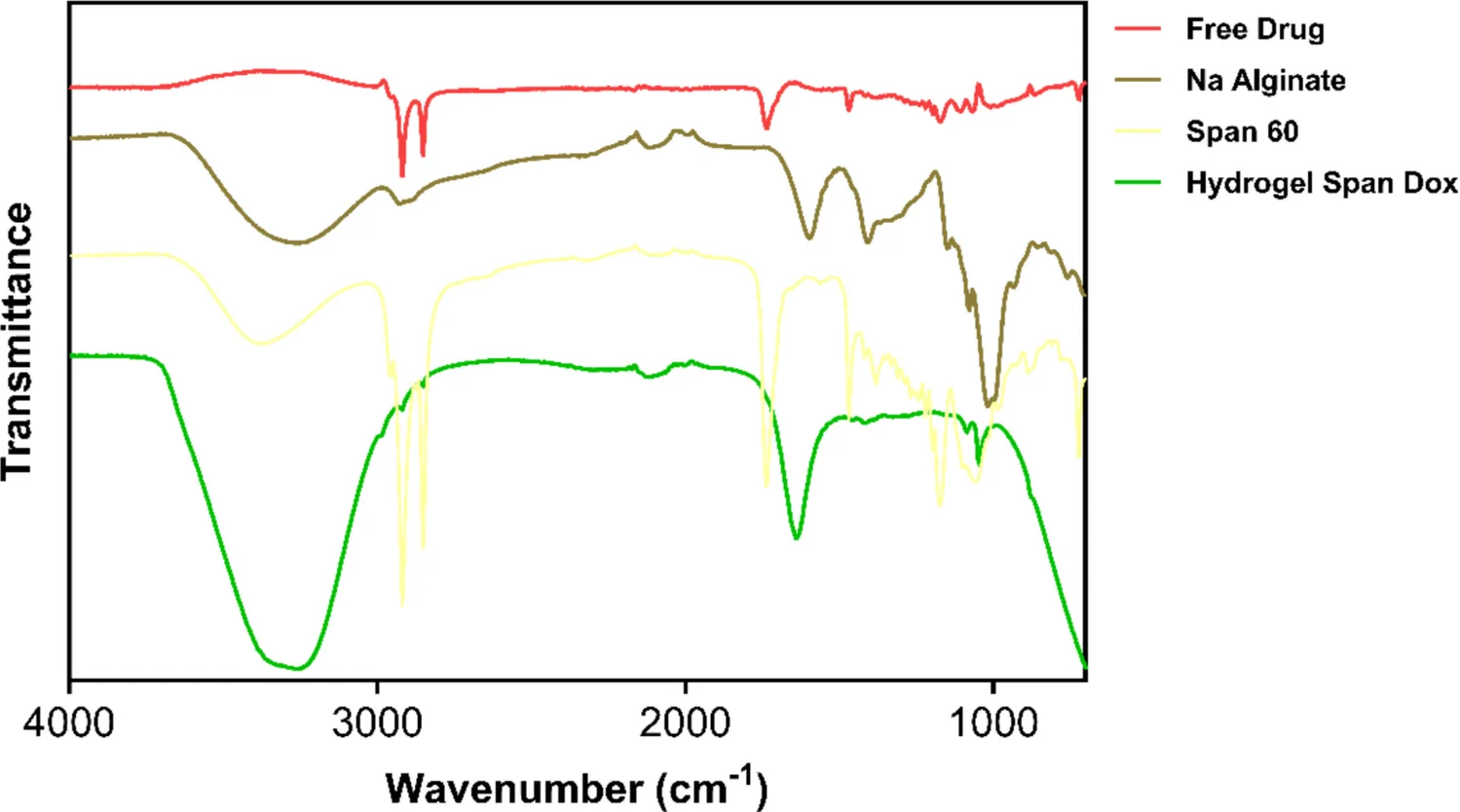
DSC spectra showing an overlay of the thermogram for free drug Doxorubicin, Span-Dox, physical mixture (Span, Doxorubicin, and Sodium alginate), and 5% alginate hydrogel loaded with Span-Dox.

DSC distinguished free doxorubicin from the carrier-based systems as seen in Fig. 5. Free doxorubicin showed a prominent endothermic event at approximately 219.8°C, consistent with a crystalline melting transition. In Span-Dox, this high-temperature drug endotherm was not detected; instead, a lower-temperature endotherm was observed at approximately 52.4°C (mean of two runs: 52.52 and 52.27°C) with a modest enthalpy (ΔH: − 11.1). The drug-loaded hydrogel system exhibited a similar low-temperature endotherm at approximately 54.35°C with a further reduction in area (ΔH: − 4.74), and no additional high-temperature endotherm attributable to crystalline doxorubicin. These thermograms indicate the loss of the free-drug melting signal upon loading and the persistence of a single low-temperature transition in the printed matrix.

#### SEM

**Fig. 6:**
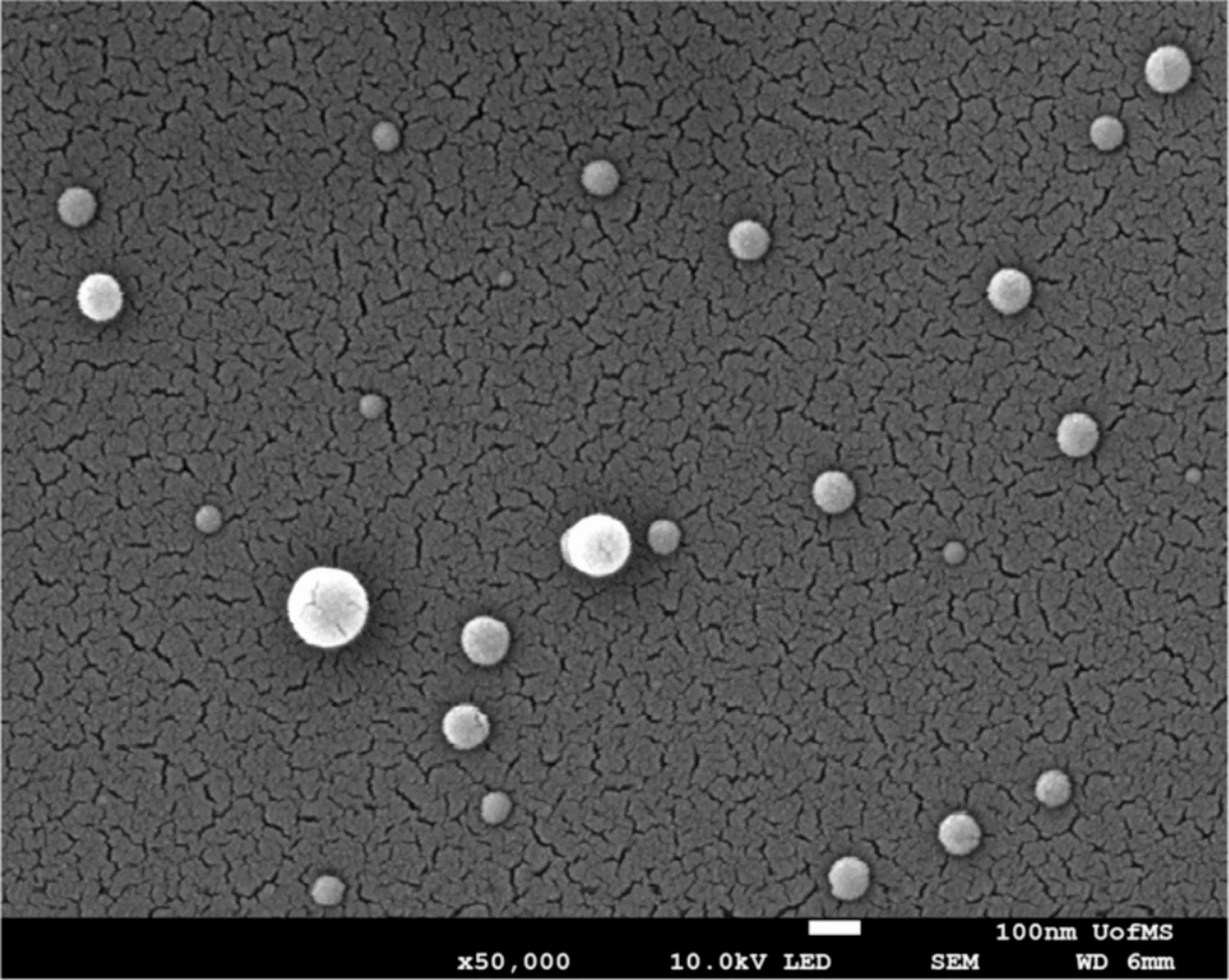
SEM image of Span-Dox at 50,000 × magnification.

The SEM characterization of the nanoparticles (Span-Dox) at 50,000 × shows a narrow to moderately distributed, discrete, predominantly spherical particles with a particle size of around 200—300 nm, as shown in Fig. 6. Surfaces appeared smooth to slightly textured with no obvious faceting. Particles were well separated with only occasional doublets, and there was no field-wide evidence of large aggregates. The cracked background pattern reflects the dried film and not particle structure.

### Drug Release

**Fig. 7:**
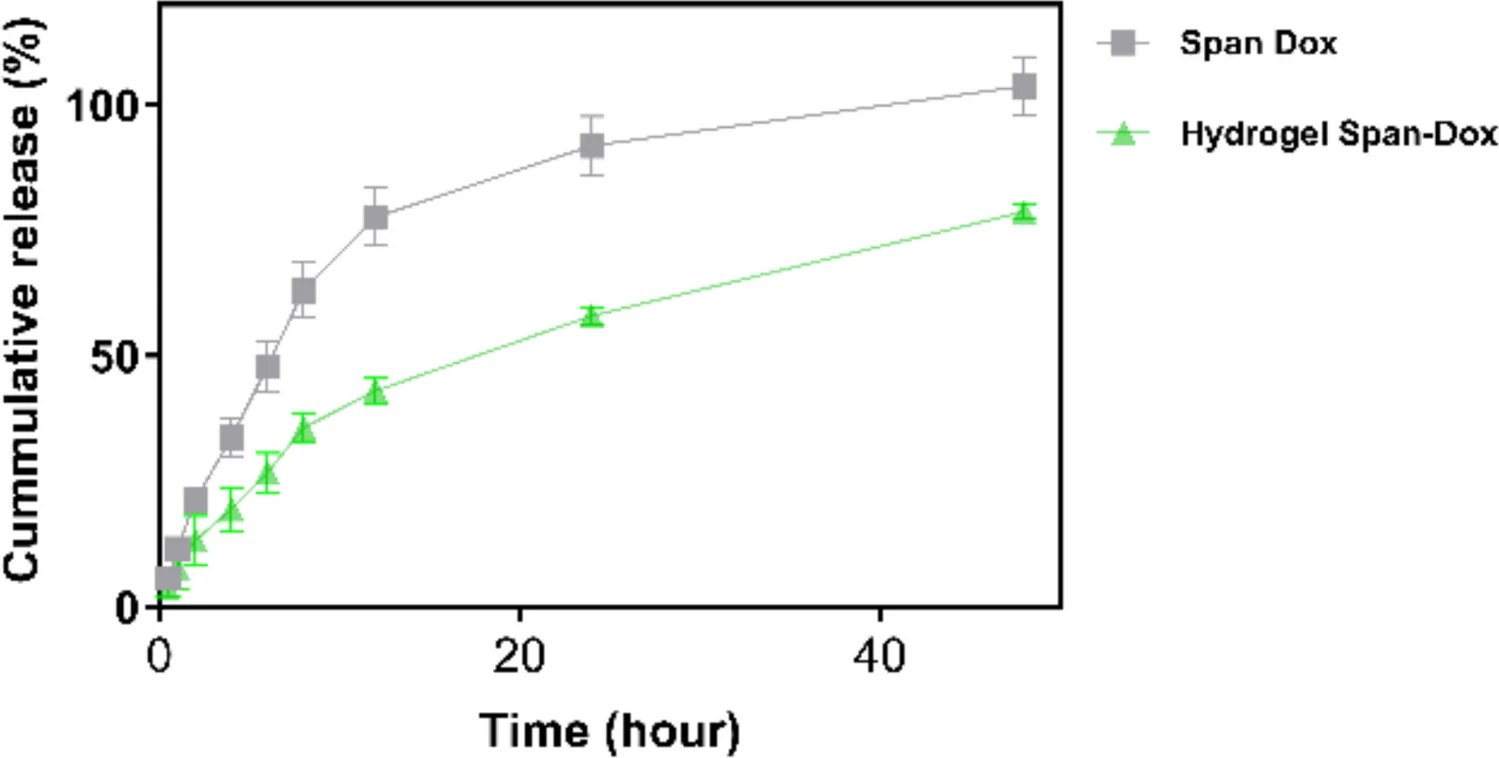
Cumulative drug release of doxorubicin from Span-Dox and FRESH 3D printed hydrogel Span-Dox in PBS at pH 7.4. The data are represented as mean ± standard deviation for *n* = 3 samples. The significance of the difference between the groups was determined using one-way ANOVA with *p < 0.05.

The drug release of Span-Dox particles and FRESH 3D printed hydrogel Span-Dox in PBS at pH 7.4 showed a cumulative release of Doxorubicin. Span-Dox showed a more rapid release than the FRESH 3D printed hydrogel Span-Dox across all time points. An initial burst was seen for Span-Dox during the first hours, followed by a slower phase that approached completion by 48 h. The FRESH-printed alginate depots containing Span-Dox showed a smaller burst and a sustained profile. By 8 h, release reached roughly one-third to one-half of the total, and by 48 h remained below full release, consistent with continued diffusion from the matrix. Mean values are plotted with standard-deviation error bars from triplicate measurements (Fig. 7).

### Cell Uptake and Cytotoxicity

**Fig. 8:**
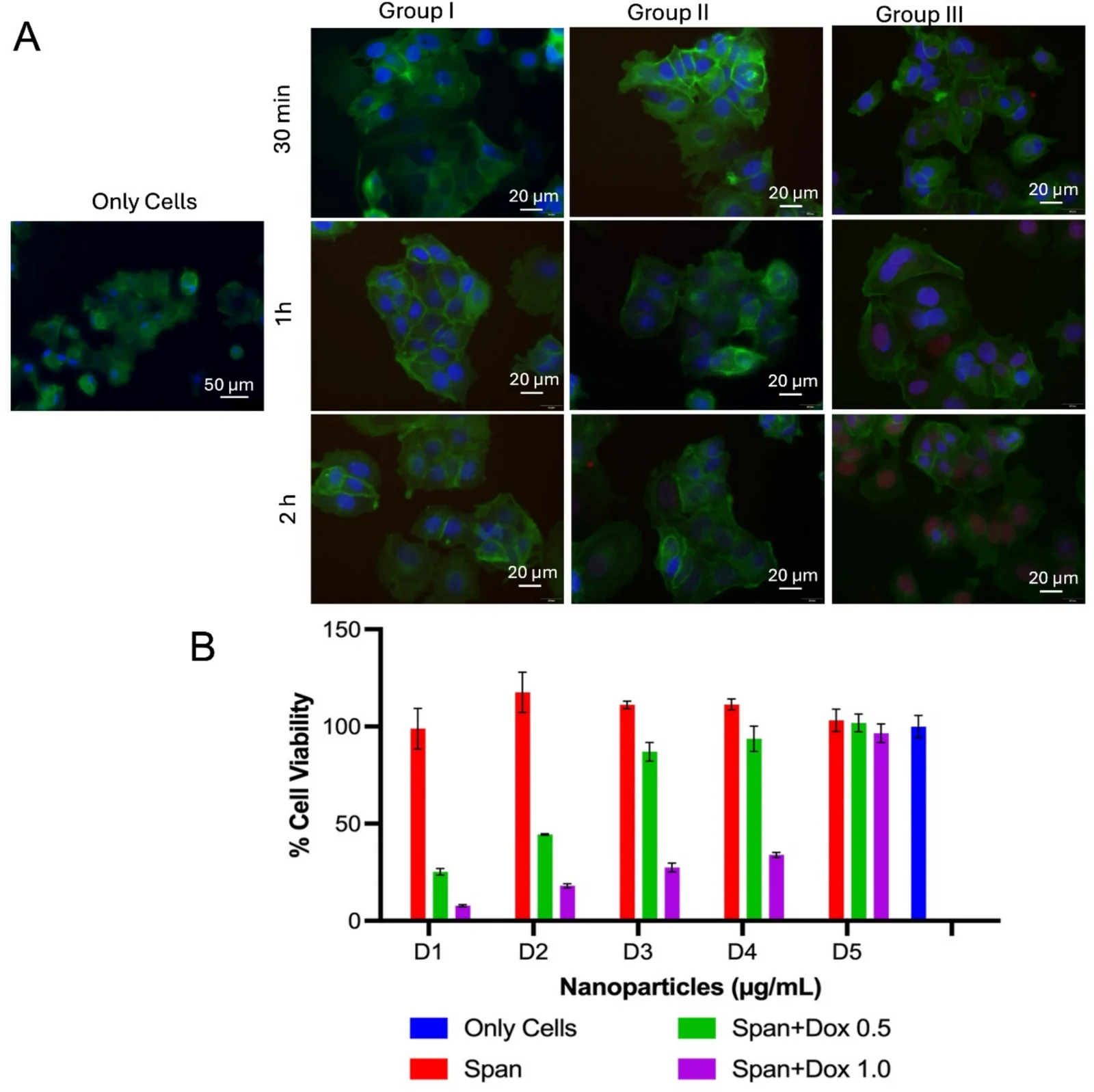
The *in vitro *analysis of Span and Span-Dox0.5&1.0 nanoparticles in MCF7 for (**A**) Cell uptake for 2 h showing the presence of Dox (red color) in the cells stained for actin filaments (green) and nucleus (blue). The red color is not significant in Span-Dox0.5 but is evident by 2 h in the Span-Dox1.0 group. (**B**)The percentage cell viability of the Span and Span-Dox0.5&1.0 nanoparticles in MCF7 cells after 24 h incubation showed a dose-dependent viability. The Span-Dox1.0 showed superior cell death and the least cell viability compared to Span-Dox0.5 and Span nanoparticles. The data are represented as mean ± standard deviation for *n* = 3 samples. The significance of the difference between the groups was determined using one-way ANOVA with *p < 0.05.

The cell uptake of the Span-Dox nanoparticles was verified in MCF7 breast cancer cells as seen in Fig. 8. The Span particles without any drug were used as a negative control, with no finding of the drug in the cells, but as a control to see the cell morphology. The cells look healthy and are similar to only cells group, signifying that the Span particles are non-toxic to cells (Fig. 3A&B). The Span-Dox particles in Fig. 8A showed a dose-dependent uptake for 2 h in MCF7 cells, where the red colour for the Dox is evident in the Span-Dox1.0 groups compared to the Span-Dox0.5 group by 2 h, respectively.

**Fig. 9:**
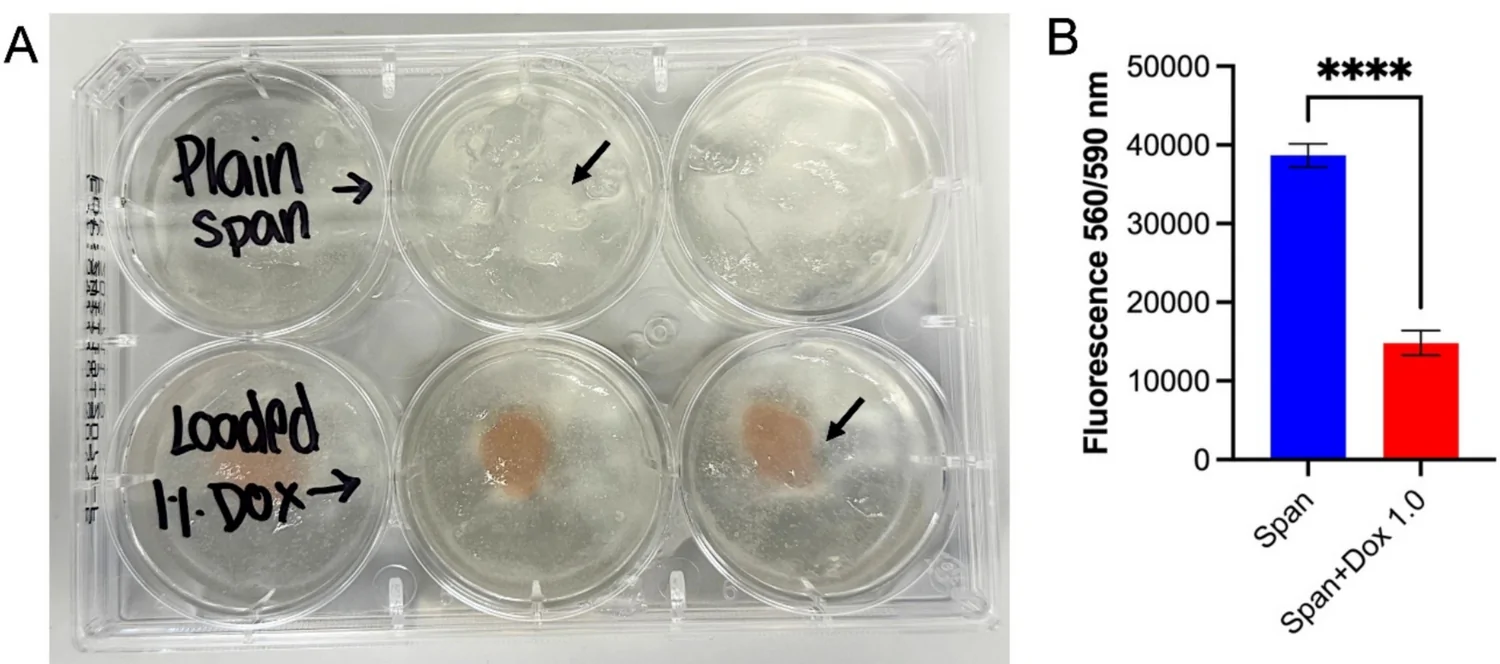
The *in vitro* transwell assay of the Span and Span-Dox1.0 nanoparticles (**A**) 3D printed alginate hydrogels by FRESH printing (Black color arrows point the FRESH printed hydrogel loaded without and with Dox loaded spanlastics in 6-well plate) and [B] Cell viability assay of the Span and Span-Dox1.0 groups in Transwell assay with MCF7 cells. The data are represented as mean ± standard deviation for *n* = 3 samples. The significance of the difference between the groups was determined using one-way ANOVA with *p < 0.05.

The FRESH printing of hydrogel was well reported in our previous study [29]. However, we would like to explore the drug-eluting behaviour of the alginate hydrogels for anticancer activity. The hydrogels loaded with the Span-Dox nanoparticles were printed, and the hydrogels maintained their shape and structure after crosslinking inside the FRESH with CaCl_2_. The anticancer activity of doxorubicin released from the printed constructs tested against MCF7 cells in a transwell assay (Fig. 8A) showed the reduction in cell viability by more than 50% (Fig. 8B). The results show that the drug loaded in the hydrogels is released from the particles and is still active to elicit a toxicity response against cancer cells *in vitro* (Fig. 9).

## Discussion

Localized drug delivery spans several platform classes, each offering distinct advantages relative to the others but with their unique limitations. Implantable polymeric depots such as the GLIADEL Wafer can achieve high local exposure at a resection site but require surgical placement and offer limited post-fabrication tunability, whereas injectable depots such as hydrogels and fibrous matrices, such as electrospun systems, reduce procedural burden but can exhibit release and persistence that depend strongly on material chemistry and the *in vivo* microenvironment [30, 31]. 3D printed drug-eluting implants extend this space by enabling patient-specific geometry and controlled release, although elution is sensitive to print architecture, including porosity and infill, and manufacturing can resemble device fabrication with frequent reliance on implantation [32–34]. Within this landscape, the present system is positioned as a localized depot that integrates spanlastic vesicles within a FRESH printed alginate hydrogel implant to couple efficient cellular association and uptake with matrix-governed sustained release at the target site, and the findings of this study support this combined formulation and manufacturing strategy for localized implant-based delivery.

From this study, it can be seen that the combination of spanlastic encapsulation and FRESH-printed alginate provides a practical route to couple efficient cellular uptake with controlled extracellular release at the target site paving way for an effective localized delivery treatment. The dose-dependent intracellular signal and the preferential nuclear localization are consistent with the known uptake and intercalative mechanism of doxorubicin and support efficient association of the Span-Dox vesicles with MCF-7 cells under serum-containing conditions. The lack of morphological changes and the high viability in the Span-only control are in line with reports that nonionic surfactant vesicles based on Span with an edge activator can be formulated with low acute cytotoxicity to mammalian cells at comparable concentrations [10, 12, 27]. These observations validate the carrier as a suitable vehicle for intracellular delivery while minimizing off-target effects from the excipients. The hydrogel data show that FRESH-printed alginate functions as an implantable drug depot that preserves vesicle function after printing and crosslinking. The transwell assay demonstrates sustained release of active drug and a significant reduction in cancer cell viability, consistent with diffusion-controlled transport through the alginate mesh and gradual vesicle-mediated release rather than a rapid burst [35–37].

The hydrogel depots show a slower, more linear profile that fits diffusion-controlled transport through the alginate network. This reflects longer path lengths than for particles in suspension. Classical models predict this behavior. In hydrated matrices, the Higuchi square-root-of-time relation and an early-time Korsmeyer–Peppas fit with *n* < 0.45 indicates Fickian diffusion rather than erosion or polymer relaxation [35, 36, 38]. The printed depots also show a smaller burst. Confinement within elastic vesicles and dispersion in a polymer mesh lowers the effective surface activity and increases tortuosity [10, 12, 36]. Spanlastic encapsulation can limit rapid desorption and flatten concentration gradients at the gel interface, which explains the early separation between the Span Dox and hydrogel curves [10, 12]. These findings agree with reports that alginate mesh size and chain entanglement regulate diffusivity, and that spanlastic or related nonionic vesicles suppress crystalline drug signals and reduce burst while maintaining payload stability [10, 12, 36, 37]. The FRESH printed, hydrogel Span-Dox converts a fast-releasing suspension into a potential implantable reservoir with geometry and matrix-controlled sustained delivery.

The current study establishes an *in vitro* proof of concept for a spanlastic-loaded, FRESH-printed alginate hydrogel implant. However, *in vivo* validation will be essential to contextualize clinical relevance and define translational boundaries. Next steps planned include: (i) local tolerability and preliminary safety following implantation, including clinical observation, local tissue response at the implant site, hematology/clinical chemistry, and histopathology of major organs; (ii) *in vivo* retention and biodistribution, to quantify drug levels at the implant site *versus* systemic exposure and to assess carrier persistence and drug retention under physiological conditions (for instance, tracking the carrier with a suitable label where appropriate); and (iii) efficacy evaluation in a relevant breast cancer model using endpoints aligned with localized therapy, such as tumor growth inhibition and histological markers of proliferation/apoptosis), supported by local toxicity and inflammation readouts. These studies will also inform implant geometry, dose loading, and administration frequency, as well as manufacturing and quality controls needed for scale-up and reproducible performance.

Encapsulation of doxorubicin in spanlastic carriers produced moderate efficiencies across the design space (33–44%), which aligns with reports for amphipathic drugs in ultra-deformable, nonionic vesicles, where partitioning into the aqueous phase limits loading [10–12]. The selected composition (Dox1) sat at the upper end of this range, suggesting that the Span 60 to Tween 80 balance and solvent-injection conditions favored tight bilayer packing and minimized early leakage during formation [11, 12, 15]. Colloidal stability, evidenced by consistent size over time and zeta potentials in the expected window for Span/Tween systems, is consistent with steric stabilization dominating over electrostatics and supports retention of the payload during storage and handling [12, 16]. Incorporation into 5% alginate did not reduce EE% relative to the suspension state, indicating compatibility of the vesicles with the hydrogel matrix and minimal drug displacement during mixing, a behavior noted for nonionic vesicles under mild conditions [11, 12]. Maintenance of EE% after FRESH printing further indicates that the gentle, embedded-printing workflow preserved vesicle integrity and limited process-induced leakage. Although the encapsulation efficiency observed here is within commonly reported ranges for spanlastic vesicles, further optimization to increase payload and minimize drug loss remains feasible. In this context, EE may be improved by tuning drug-to-surfactant ratio and vesicle composition (thus, Span/Tween ratio, edge activator type and concentration, and inclusion of membrane-stabilizing components where appropriate) to enhance drug-membrane association and reduce leakage. Process parameters such as hydration conditions, temperature, mixing energy, and solvent removal rate can also influence vesicle integrity and drug retention. In addition, the apparent EE can be affected by the separation method used to remove unentrapped drug, in this study, centrifugation (10,000 rpm, 15 min, 4 °C) may contribute to recovery losses and/or leakage during sedimentation and handling of deformable vesicles. Scalable alternatives include ultrafiltration/centrifugal filtration, tangential flow filtration, dialysis, or size-exclusion chromatography which may reduce separation-associated losses, with trade-offs in throughput and recovery. Total drug recovery of about 40% following ultracentrifugation point to separation losses rather than chemical instability; switching to size-exclusion or ultrafiltration and routine mass-balance checks can reduce losses and tighten confidence intervals on EE% [15, 16]. The data support a stable, formulation-dependent encapsulation that withstands hydrogel incorporation and printing, providing a sound basis for the sustained-release and efficacy studies that follow.

Altering the Span 60 to Tween 80 ratio and the phase in which Tween 80 was introduced modulated vesicle formation in a manner consistent with the role of edge activators in spanlastic systems. Ethanol injection reliably produced vesicles in the nanometer range, and the choice of placing Tween 80 in either the organic or aqueous phase influenced interfacial mixing and membrane curvature during nucleation, which is known to affect final size and polydispersity [10, 12, 15]. The formulation that was advanced for loading was selected based on particle size and reproducible pellet formation after centrifugation, a practical indicator of vesicle yield for non-ionic systems prepared by solvent injection [15, 16]. Beyond these metrics, the Span 60 to Tween 80 balance also has implications for biological performance, since Span 60 promotes tighter membrane packing and drug retention, whereas Tween 80 increases membrane elasticity and can enhance vesicle interaction with biological interfaces and cellular association [10, 12, 14]. The mean diameter of approximately 300 nm remained stable over eleven days, indicating physical stability of the dispersions prepared under these conditions and robustness of the process window for spanlastics [10, 14, 16]. Zeta potentials within the range reported for Span–Tween vesicles suggest that colloidal stability here is provided primarily by steric contributions from non-ionic surfactants rather than by strong electrostatic repulsion, which aligns with prior reports on spanlastic and niosomal carriers [10, 12, 14]. Drug inputs of 0.5 and 1.0 percent w/v did not disrupt size control in the selected batches [10, 14]. Together, these observations support ethanol injection as a robust manufacturing route and identify a composition suitable for incorporation into alginate inks, providing a basis for linking formulation to printing performance and release behavior. For applications that rely on systemic transport, further reduction of size may be desirable, but in the context of local, FRESH-printed depots, the observed stability and nanometer-scale dimensions are appropriate for sustained release from the hydrogel matrix [10, 14].

The particles in SEM appear mostly spherical with smooth surfaces, consistent with vesicles formed from Span 60 with an edge activator and supporting successful assembly of Span Dox [10, 12]. No extensive flocculation was observed, which suggests that the surfactant composition and surface charge were sufficient to maintain colloidal stability during drying [10, 12, 27]. Occasional doublets likely reflect minor coalescence on the substrate rather than instability in bulk suspension. The visible spread in sizes matches the PDI measured by DLS and is expected when comparing hydrated measurements with images of dried specimens. Because SEM captures dried particles, absolute diameters can be smaller than hydrodynamic sizes from DLS, so the image chiefly confirms particle shape, surface quality, and the absence of large aggregates [10, 12, 27]. The SEM confirms DLS results, indicating well-dispersed spanlastic vesicles suitable for incorporation into the hydrogel matrix and for reproducible drug loading and release.

The spectral features support successful encapsulation of doxorubicin within the spanlastic carriers and its compatible incorporation into the alginate matrix. Relative to free doxorubicin, broadening of the hydroxyl envelope and attenuation of the quinone carbonyl and aromatic bands in the Dox-loaded vesicles are consistent with strengthened hydrogen bonding and hydrophobic associations that shift the drug from a crystalline or self-associated state toward a carrier-associated environment [24, 39]. In the hydrogels, the dominance of alginate hydroxyl, carboxylate, and glycosidic bands, coupled with reduction or overlap of drug-specific signals after loading, indicates that the drug remains largely associated with vesicles dispersed in the polymer network and that vesicle–polymer interactions are primarily noncovalent rather than covalent [25, 27, 28]. This interpretation aligns with commonly reported FTIR signatures of drug encapsulation in niosomal systems, where masking or downshifts of carbonyl/aromatic bands accompany carrier association without the appearance of new functional groups [27, 28]. Concurrence with DSC, specifically depression or loss of the drug’s melting endotherm, further support amorphization or confinement within the carrier and gel microenvironment [40].

These findings align with prior work indicating that alginate concentration and network structure govern diffusivity and that embedded printing supports high-fidelity constructs suitable for localized delivery [18, 19, 29, 37].

## Conclusion

The Span-Dox nanoparticles were prepared in the range of 200–300 nm, which facilitate cancer cellular uptake. The findings demonstrated the effective loading of Dox into Spanlastics, providing a consistent drug release. The FRESH 3D printing of hydrogels construct with Span-Dox nanoparticles facilitated the modulation of drug release profiles and enhanced cytotoxic efficacy against MCF7 cancerous cells *in vitro*. Together, these findings highlight the promise of Spanlastics 3D‑printed hydrogel constructs as a tunable and potent cancer‑therapeutic platform, paving the way for future *in vivo* studies to advance their potential translational impact in targeted antitumor treatment.

## Data Availability

The data is available upon request from the corresponding author.
